# Treatment strategy for heart failure complicated with complete left bundle branch block and atrial tachycardia: a case report

**DOI:** 10.1186/s13256-024-04343-3

**Published:** 2024-01-27

**Authors:** Jian Wang, Qing-Qing Zhang, Jie Lin, Wei Yuan, Li-Chun Wang, Yu-Cheng Wu

**Affiliations:** 1 Department of Cardiology, The Affiliated Taizhou People’s Hospital of Nanjing Medical University, Taizhou School of Clinical Medicine, Nanjing Medical University, No. 366 Taihu Road, Taizhou, 225300 Jiangsu Province China; 2 Department of Endocrinology, The Affiliated Taizhou People’s Hospital of Nanjing Medical University, Taizhou School of Clinical Medicine, Nanjing Medical University, Taizhou, 225300 Jiangsu Province China

**Keywords:** Heart failure, Complete left bundle branch block, Left bundle branch area pacing, Atrial tachycardia, Case report

## Abstract

**Background:**

For patients with heart failure combined with complete left bundle branch block, cardiac resynchronization therapy is an important therapeutic method. If these patients also have atrial tachycardia, how to choose a treatment strategy deserves discussion.

**Case presentation:**

A Chinese woman in her early 70s was admitted due to recurrent episodes of chest distress and asthma for 1 year. Physical and laboratory examinations showed filling of the jugular vein, lung rales, left enlargement of the heart boundary, edema of the lower limbs and elevation of N-terminal pro b-type natriuretic peptide. An electrocardiogram showed atrial tachycardia and a left bundle branch block. An echocardiography revealed enlargement of the left ventricle and left ventricular systolic dysfunction. After obtaining informed consent, the treatment strategy decided upon by the team was to use biventricular cardiac resynchronization therapy treatment and to not intervene for the atrial tachycardia, with left bundle branch area pacing as a backup. Due to twisted and narrow coronary vein branches, traditional biventricular pacing failed, and then, left bundle branch area pacing was attempted successfully. A follow-up echocardiography at 1 year showed improved systolic function. The outcomes for this patient are favorable, but the choice of interventional strategy is worthy of discussion in this case.

**Conclusion:**

For patients with heart failure combined with left bundle branch block and atrial tachycardia, left bundle branch area pacing can replace traditional biventricular pacing for cardiac resynchronization therapy treatment, and the therapeutic effect is significant. However, multiple factors need to be considered when formulating strategies, including whether there is bundle branch block under sinus rhythm, the success and recurrence rate of atrial tachycardia ablation, the response of cardiac resynchronization therapy, the costs of different strategies, and instrument implantation issues.

## Background

Cardiac resynchronization therapy (CRT) is an important treatment method for heart failure patients with left ventricular systolic dysfunction and conduction system abnormalities [1]. The traditional implantation method of CRT is mainly biventricular pacing. Recently, physiological pacing, such as His bundle pacing (HBP) and left bundle branch area pacing (LBBAP), has been increasingly used in CRT [2–4]. Studies have shown that in nonischemic heart failure patients, the success rate of LBBP implantation is between 81% and 97%, the QRS duration is shortened by more than 50 ms [5], left ventricular ejection fraction (LVEF) is increased by 37% ± 12% [5] and 55% ± 10% [6], heart function is significantly improved, and the left ventricular end-diastolic diameter (LVEDD) is shortened by 59 ± 9 mm [5], effectively achieving cardiac resynchronization therapy and reversing left ventricular remodeling. Further research is needed on the preferred treatment method in clinical practice. Interestingly, if the above-mentioned patients are complicated with atrial tachycardia, the selection of an interventional treatment strategy and the role of atrial tachycardia ablation in the strategy have not been reported in the literature. Here, we report a patient’s treatment strategy for heart failure, complete left bundle branch block (LBBB), and atrial tachycardia (AT).

## Case presentation

A 73-year-old Chinese woman was admitted to our institution due to recurrent episodes of chest distress and asthma for 1 year, which had worsened over the last month. She had no history of hypertension, diabetes, or chronic bronchitis. The patient’s temperature was 36.6 °C, heart rate was 111 bpm, respiratory rate was 18 breaths *per* minute, blood pressure was 125/70 mmHg and oxygen saturation was 98% in room air. There was filling of the jugular vein, lung rales, left enlargement of the heart boundary, and edema of the lower limbs.

On admission, her blood tests, including routine blood tests, renal function, liver function, thyroid function, and coagulation function showed no abnormalities. The level of N-terminal pro b-type natriuretic peptide (NT-proBNP) was 4472.3 ng/L on admission. Chest computed tomography (CT) indicated pulmonary infection with pleural effusion on both sides. Electrocardiogram (ECG) showed atrial tachycardia and LBBB (QRS duration 130 ms). Echocardiography revealed an enlarged heart (LVEDD 52 mm), and left ventricular systolic dysfunction (LVEF 34%). There were no obvious abnormalities on abdominal color Doppler ultrasound. Coronary angiography revealed 30% stenosis in the proximal and middle segments of the left anterior descending branch, and no apparent stenosis was observed in the remaining coronary arteries.

The patient’s final diagnosis was as follows: heart failure, dilated cardiomyopathy, New York Heart Association (NYHA) grade III, LBBB, atrial tachycardia, coronary atherosclerosis, pulmonary infection, and pleural effusion. The medications, which were started at three months before admission, were as follows: metoprolol 47.5 mg orally (po) once a day (qd), dapaglifoxin 10 mg po qd, sacubitril valsartan 50 mg po bid, torsemide 10 mg po qd, and spironolactone 20 mg po qd. The additional medications started after admission included digoxin 0.125 mg po qd, recombinant human brain natriuretic peptide (which was given for 3 days), intravenous diuretics that were given intermittently, and cefodizime sodium 2 g intravenous glucose tolerance test (ivgtt) twice a day (bid). After 10 days of admission treatment, the patient still had symptoms of heart failure, so interventional treatment was recommended.

**Fig. 1:**
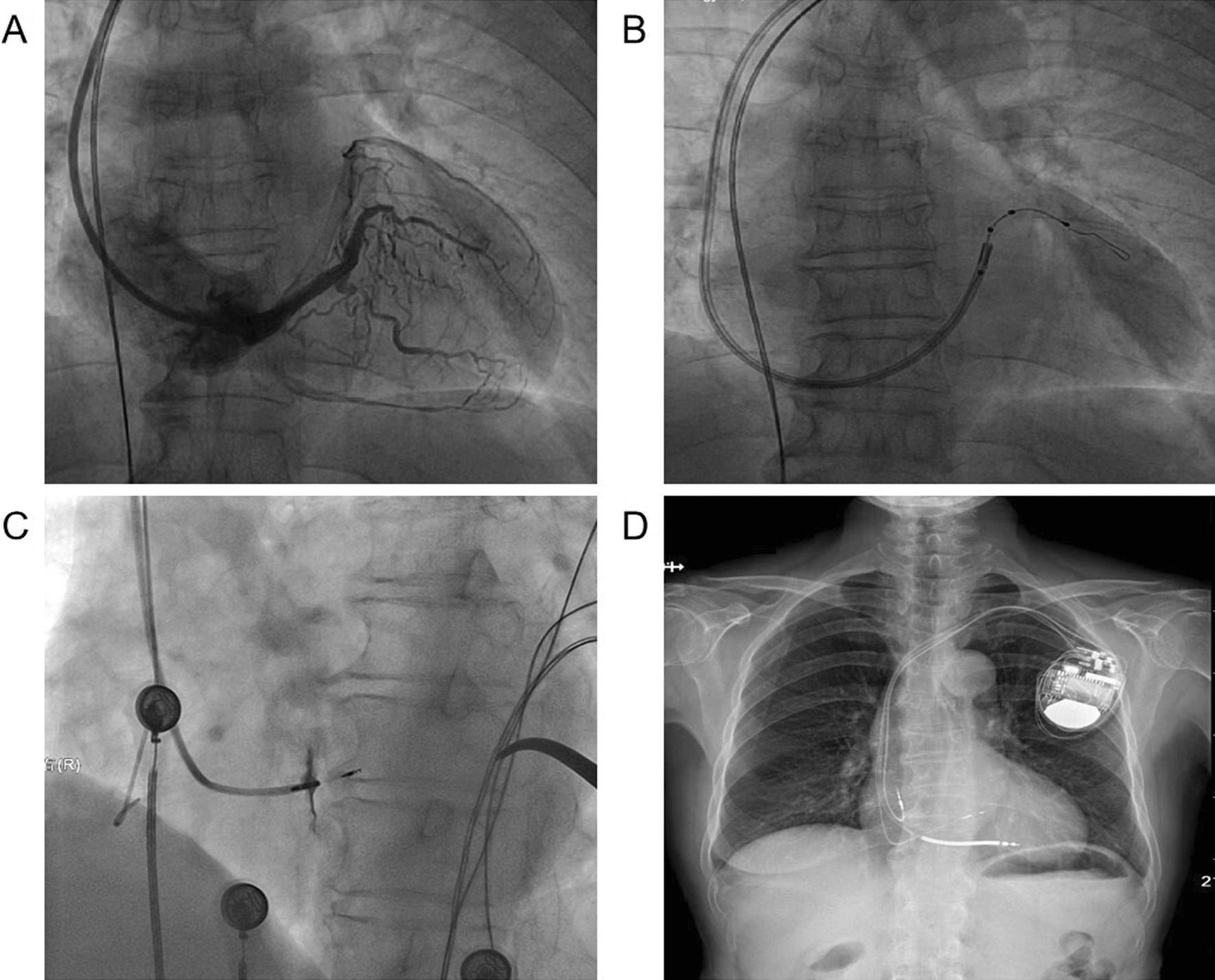
X-ray imaging of the interventional procedure process. **A** The coronary vein with severe distortion of the lateral posterior vein and a narrow and tortuous proximal posterior vein. **B** The left ventricular lead could not reach the target vessel. **C** Angiography of 3830 electrode in interventricular septum. **D** Final imaging after pacemaker implantation

The interventional treatment strategy included biventricular CRT treatment with LBBAP as a backup. For atrial tachycardia, the medications were given without radiofrequency ablation. Under local anesthesia, a steel wire was inserted into the inferior vena cava through the left subclavian vein. An electrode was inserted into the coronary vein sinus through the sheath, and then a long sheath was sent into the coronary sinus. Coronary sinus imaging showed that the coronary vein was normal in shape, but there was severe distortion of the lateral posterior vein and a narrow and tortuous proximal posterior vein (Fig. 1A). Attempts were made to select the lateral posterior vein and posterior vein as the target vessels, but under the guidance of the PTCA wire, the left ventricular lead could not reach the distal segment of the target vessel for fixation (Fig. 1B).

After discussion, we decided to try LBBAP instead of the original plan, and the patient agreed. A C315 His sheath was inserted along the guide wire, and then the His potential was detected with the 3830 electrode. The operator moved the sheath toward the apex direction approximately 1 cm, and rotated the electrode while testing the parameters. Finally, a QRS wave in the right bundle branch delay was obtained with satisfactory parameters (threshold 0.6 V, R amplitude 13.7 mv, impedance 815 Ω). Afterward, the right atrial electrode and the right ventricular defibrillation electrode were implanted separately (Fig. 1C, D). Each pacing lead was fixed and connected to the cardiac resynchronization therapy defibrillator (CRT-D) pulse generator (Medtronic DTBC2D1), and the parameters of the CRT-D test were acceptable.

**Fig. 2:**
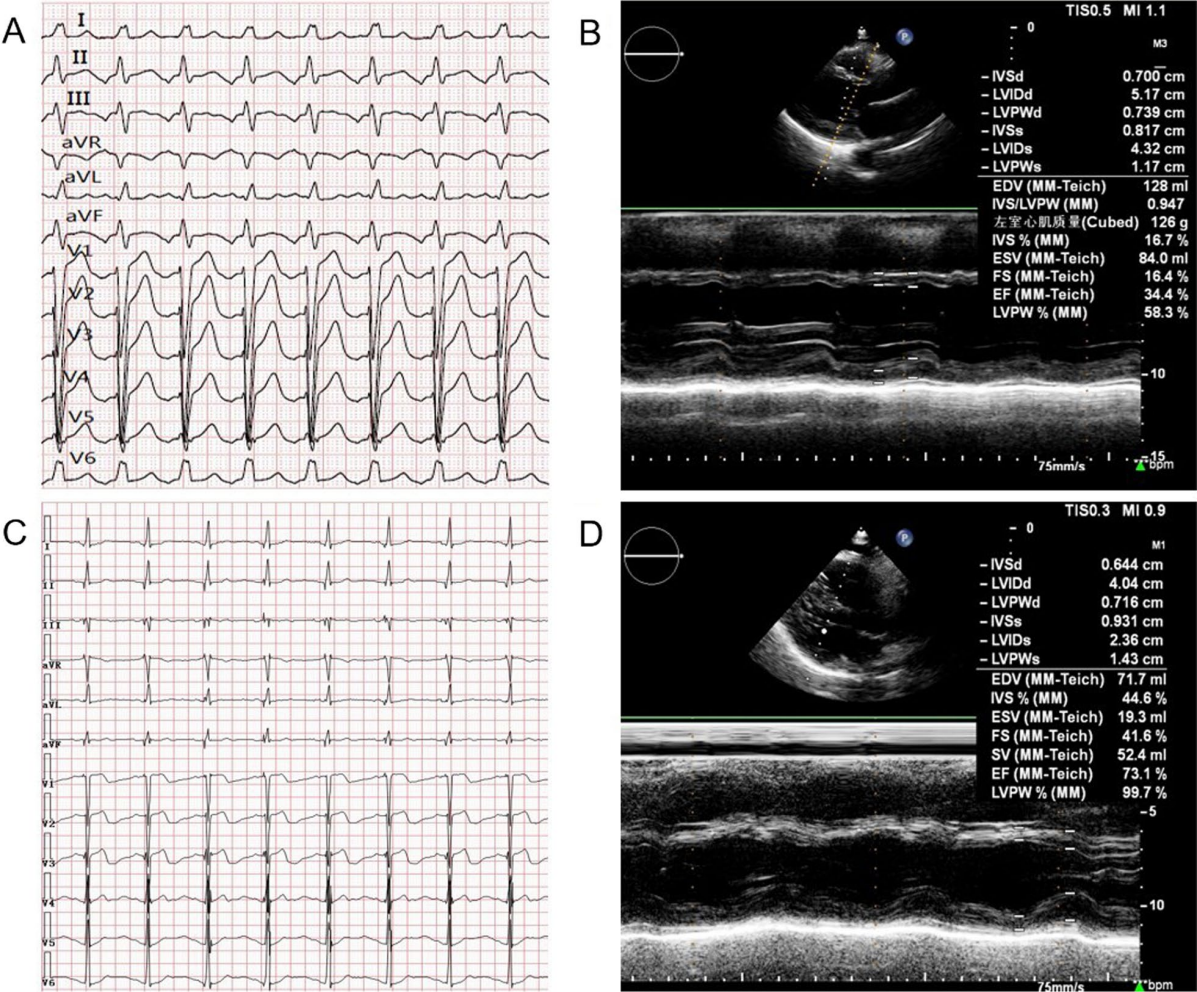
Comparison of preoperative and postoperative electrocardiogram and echocardiography. **A** Preoperative ECG showed atrial tachycardia and LBBB (QRSd 130 ms). **B** Preoperative echocardiography showed left ventricular dilatation (LVEF 34.4%). **C** Postoperative ECG showed sinus rhythm with ventricular pacing (QRSd 70 ms). **D** 1-year follow-up echocardiography showed left ventricular retraction (LVEF 73.1%)

Postoperative ECG showed sinus rhythm, atrial perception, and ventricular pacing. The postoperative QRS duration was 70 ms, which was significantly narrower than the preoperative QRS duration (Fig. 2A, C). The patient was continuously treated with drugs following the doctor’s advice. Follow-up echocardiography was performed at 1 week, 1 month, 3 months, and 1 year (Table 1) and showed that the LVEF had increased remarkably and that the left ventricle had obviously retracted (Fig. 2B, D). Atrial tachycardia was controlled satisfactorily with metoprolol. The patient’s heart failure symptoms significantly improved, the NYHA cardiac function was grade I, and the patient’s daily physical activity was not limited.

**Table 1:** Echocardiography results of admission and follow-up

Time	LAD (mm)	LVEDD (mm)	LVESD (mm)	LVEF (%)
Admission	46	52	43	34
1 week postoperation	32	48	39	37
1 month postoperation	35	47	37	48
3 month postoperation	39	45	30	63
12 month postoperation	33	40	24	73

*LAD* left atrial diameter, *LVEDD* left ventricular end diastolic diameter, *LVESD* left ventricular end systolic diameter, *LVEF* left ventricular ejection fraction

## Discussion and conclusion

Here, we report a patient with heart failure, LBBB, and atrial tachycardia who underwent cardiac resynchronization treatment using LBBAP, with satisfactory follow-up results. The postoperative QRS duration was shortened to 70 ms, the LVEF had increased to 73%, and the LVEDD had decreased to 40 mm at the 1-year follow-up. The heart failure symptoms were significantly improved by effectively reversing the left ventricular remodeling. But in this case, what is worth discussing is not the outcome but the choice of treatment strategy.

The preferred treatment strategy is atrial tachycardia radiofrequency ablation, CRT, or both. If atrial tachycardia ablation is chosen, the possible scenarios are as follows: disappearance of atrial tachycardia, disappearance of LBBB under sinus rhythm, simultaneous resolution of ventricular asynchrony, and no instrument implantation. This result is the best, but the success rate of ablation and the risk of atrial tachycardia recurrence should be considered [7]. Another scenario is the disappearance of atrial tachycardia, while remaining LBBB under sinus rhythm and cardiac asynchrony are still present. At this point, it may be necessary to continue and complete CRT. In this situation, the cost, time, and risk of operation need to be weighed. If CRT is chosen and if the postoperative cardiac synchronization is good, then the control effect of drug on atrial tachycardia is also fine. This result is also very good; however, there are also some issues, such as ineffective response to cardiac resynchronization, high costs, the need for regular pacemaker replacement, and poor control of the atrial tachycardia with drugs leading to recurrent tachyarrhythmia [8].

Therefore, the authors suggest that a better treatment strategy than that of this case is as follows: first, attempt cardioversion with overspeed suppression or medication during the procedure; then, if the LBBB disappears under sinus rhythm, choose the radiofrequency ablation plan for atrial tachycardia; and if the LBBB remains after cardioversion, choose the CRT plan.

There is still controversy over whether to choose biventricular pacing or physiological pacing (HBP or LBBAP) for CRT. In clinical practice, the most common problem with biventricular CRT is the low effective response rate. In addition, factors such as twisted and narrow coronary veins may lead to failure of left ventricular electrode implantation [9]. Increasing evidence suggests that LBBAP can significantly shorten the QRS duration, increase the LVEF, and improve clinical symptoms and prognosis [10]. At present, LBBAP is a safe and effective alternative to biventricular CRT, and whether it can be used as the main treatment method still requires multicenter randomized controlled studies and further evaluation of long-term prognosis [11]. Currently, the two methods can be alternative options to each other, but LBBAP may become the preferred treatment strategy for CRT in the future.

For patients with heart failure combined with LBBB and atrial tachycardia, LBBAP can replace traditional biventricular pacing for CRT treatment, and the therapeutic effect is significant. However, when selecting interventional treatment strategies for such patients, multiple factors need to be comprehensively considered, including whether there is bundle branch block under sinus rhythm, the success rate and recurrence rate of atrial tachycardia ablation, the response of CRT, the operator’s experience, the costs of the different strategies, and the instrument implantation issues.

## Data Availability

The datasets used and/or analyzed during the current study are available from the corresponding author on reasonable request.
